# Angiogenesis in the Avian Embryo Chorioallantoic Membrane: A Perspective on Research Trends and a Case Study on Toxicant Vascular Effects

**DOI:** 10.3390/jcdd7040056

**Published:** 2020-12-05

**Authors:** Warren Burggren, Maria Rojas Antich

**Affiliations:** Developmental Integrative Biology Group, Department of Biological Sciences, University of North Texas, Denton, TX 76203, USA; maria.rojas@unt.edu

**Keywords:** angiogenesis, chicken embryo, chorioallantoic membrane, crude oil

## Abstract

The chorioallantoic membrane (CAM) of the avian embryo is an intrinsically interesting gas exchange and osmoregulation organ. Beyond study by comparative biologists, however, the CAM vascular bed has been the focus of translational studies by cardiovascular life scientists interested in the CAM as a model for probing angiogenesis, heart development, and physiological functions. In this perspective article, we consider areas of cardiovascular research that have benefited from studies of the CAM, including the themes of investigation of the CAM’s hemodynamic influence on heart and central vessel development, use of the CAM as a model vascular bed for studying angiogenesis, and the CAM as an assay tool. A case study on CAM vascularization effects of very low doses of crude oil as a toxicant is also presented that embraces some of these themes, showing the induction of subtle changes in the pattern of the CAM vasculature growth that are not readily observed by standard vascular assessment methodologies. We conclude by raising several questions in the area of CAM research, including the following: (1) Do changes in patterns of CAM growth, as opposed to absolute CAM growth, have biological significance?; (2) How does the relative amount of CAM vascularization compared to the embryo per se change during development?; and (3) Is the CAM actually representative of the mammalian systemic vascular beds that it is presumed to model?

## 1. Introduction

The development of the vertebrate circulation has long been of interest to life scientists, with the earliest observations of circulatory development attributed to Aristotle’s reports on the beating heart of a chicken embryo.

“*In the case of the hen, the first signs of the embryo are seen after three days and nights …the heart is no bigger than just a small blood-spot in the white …and beats and moves as though it were alive; and from it, as it grows, two vein-like vessels with blood in them lead on a twisted course to each of the two surrounding membranes. A membrane with bloody fibers already surrounds the white of the egg, at this time coming from the vessel-like channels.*”

Aristotle, Historia Animalium.

These observations on the embryo of the chicken, *Gallus gallus domesticus*, portended a major focus on the development of the cardiovascular system of this species more than two millennia later. Indeed, a query in PubMed for “chicken” and “embryo” and “heart” yields > 1700 articles (>370 in the last decade), with many specifically using the chicken embryo as a model for investigating the circulation generally. It is beyond the scope of this article to consider the breadth of this literature, but several informative reviews of the use of the bird embryo as a model for a variety of physiological and morphological processes have been published; see [1,2,3,4,5,6] for an entry into the voluminous literature.

A common theme in many of the current studies of avian cardiovascular development is investigation of the chorioallantoic membrane (CAM), a delicate, highly vascularized membrane immediately under the egg shell that functions in oxygen uptake and carbon dioxide elimination for the developing embryo [7]. The focus on the CAM is driven by interest in enhancing our understanding of several areas of cardiovascular development and that of other systems. Many excellent reviews on the subject matter of each of these themes are available, as we indicate below. However, the goal of this perspective article is to bring together in one place the major themes of chorioallantoic research, with the hope that cross pollination between CAM-related research areas can continue.

## 2. Contemporary Themes in Chorioallantoic Membrane Investigation

Research on the chorioallantoic membrane of the chicken embryo has been conducted from several different perspectives, seeking several different outcomes. Below, we provide examples (as opposed to an exhaustive review) of studies contributing to our knowledge within the various themes of CAM research.

### 2.1. The CAM as an Intrinsically Interesting Gas Exchange and Osmoregulation Organ

**Figure 1:**
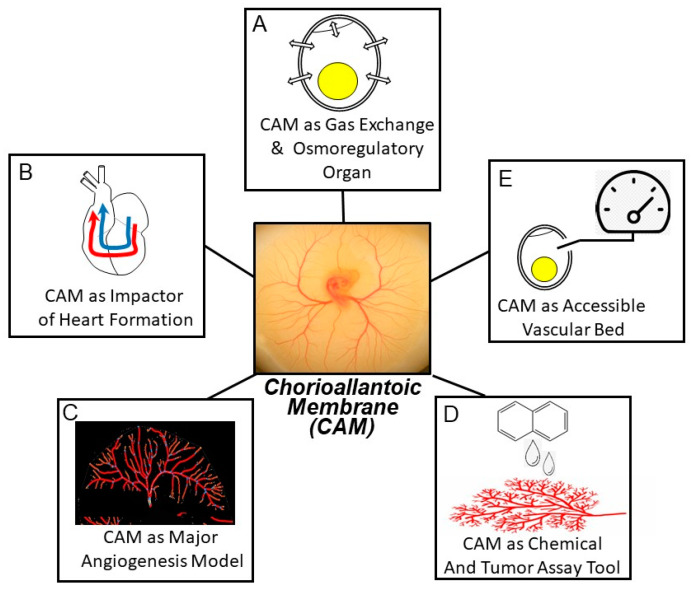
Some examples of major themes in chorioallantoic membrane (CAM) research involving respiratory gas and ion and water exchange (**A**), cardiac development (**B**), angiogenesis (**C**), toxicology and oncology (**D**), and general physiology (**E**). See text for additional discussion.

**Figure 2:**
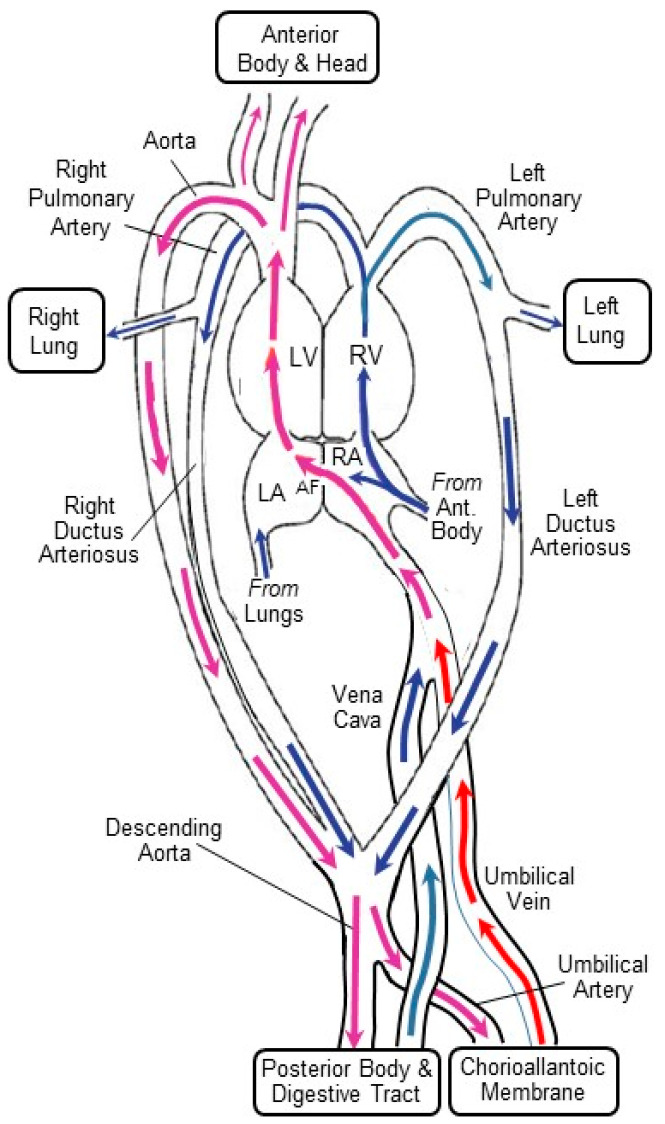
Vasculature and potential pathways of blood flow in the chicken embryo at mid-incubation. In this highly schematic depiction, cardiovascular structures, vascular confluences, and branch points are not to scale, and not all vessels are depicted or labelled. Blue and red arrows indicate flow of deoxygenated and oxygenated blood, respectively, with purple arrows depicting mixed oxygenated and deoxygenated blood resulting from intra- or extra-cardiac shunts. AF, atrial foramina; LV, left ventricle; RV, right ventricle; LA, left atrium; RA, right atrium. Modified from [13,14,15].

Most biologists familiar with the avian chorioallantoic membrane are exploiting its many favorable characteristics to study basic developmental processes, especially angiogenesis. That is, the CAM is a model for other vascular beds and other species, especially humans. Before discussing these crucial applied applications, it is important to realize that the CAM of bird embryos has been investigated for decades as an intrinsically interesting organ/vascular bed involved in gas exchange and osmoregulatory/excretory functions (Figure 1A) [7,8,9,10,11,12]. What clearly emerges from these pioneering studies is a picture of the chorioallantoic membrane as an exquisitely evolved gas exchanger. The CAM, complexly, is located both in series and in parallel with the systemic vasculature (Figure 2). This arrangement derives from two anatomical characteristics of the avian embryo: (1) the right-to-left shunt intracardiac shunt via the atrial foramen, allowing deoxygenated blood returning from the systemic vascular beds to pass from the right atrium into the left atrium and onto the left ventricle, and (2) the confluence of the chorioallantoic veins carrying oxygenated blood with the central systemic veins draining the digestive tract and anterior of the body prior to entering the right atrium.

A notable characteristic of the CAM is that this organ emerges rapidly in the first few days of development, sustaining gas exchange and growth throughout the rapid development and growth of the embryo until the embryo internally pips and transitions to pulmonary respiration. An infrequently studied part of this overall process is how the tissues of the CAM undergo rapid apoptosis and senescence as internal pipping occurs [16,17].

While the respiratory functions of the CAM have been well categorized, far less appreciated is the additional role of the avian CAM as an organ for water and ion exchange with the compartments that overlie and underlie it. As early as the 1970s, it was appreciated that active Na^+^ transport across the CAM was responsible for altering water loss across the CAM and, in turn, from the egg overall [18,19,20,21]. The CAM appears to act in concert as the kidney develops through its three phases of pro-, meso- and metanephros [22,23].

As the lungs grow during the final days of embryonic development in preparation for pulmonary gas exchange, the CAM prepares to undergo rapid senescence upon internal pipping, when the embryo’s beak penetrates into the air cell and the first breath is taken. Most of the same remarkable circulatory changes seen in mammals upon the fetus’ first breath also occur in the avian embryo as it switches to pulmonary gas exchange [24]. Not surprisingly, focus has been on the study of the establishment of the pulmonary circulation. However, the rapid degeneration and de-perfusion of the CAM, and the shift of a large volume of blood from the CAM into the embryo’s system circulation, almost surely involve hormonally induced apoptosis, and deserves additional study.

### 2.2. The CAM’s Hemodynamic Influence on the Developing Heart and Central Vessels

Another theme in our investigations of the CAM and its expanding vascular network in early development—germane to the topic of this journal’s Special Issue—is interpreting the effects that these resistance vessels will have on the preload, afterload, and shear stresses experienced by the developing heart (Figure 1B). While many aspects of heart transition from a straight tube to an S-curved tube through chamber formation are genetically dictated, it is also clear that changes in both preload (induced by unilateral clip placement on a vitelline vein) and afterload (induced by ligation of an outflow artery) can alter cardiac development (both morphologically and physiologically); for example, see [25,26,27,28,29,30,31,32,33,34,35].

#### 2.2.1. Preload

Changes in preload in the chicken embryo specifically induced by interruption of CAM venous return have been investigated by unilateral clipping of one of the major vitelline veins returning from the CAM [30,31,32,33,34] and by suffusion with sodium nitroprusside and acute venous hemorrhage [36]. These experimentally induced changes in venous return of oxygenated blood, and in the preload on the heart, lead to changes in both heart architecture [33,34] and cardiac performance [30,31,32]. Interestingly, extra-embryonic venous clipping, while having major effects on the heart, had no effect on the normal development of the extremities, head, and eyes [33]. These data suggest that the changes in cardiac architecture resulted from preload-related hemodynamic changes, rather than systemic decreases in oxygenation or nutrients caused by reduced venous return.

#### 2.2.2. Afterload

The CAM vascular bed is a major component of the embryonic circulation’s peripheral resistance, and thus a major factor in afterload imposed on the developing heart. Multiple factors influence afterload in the embryonic heart of the chicken embryo. Hypoxic exposure of the chicken embryo at ~3.5 days post-fertilization, for example, results in increased peripheral resistance and thus increased cardiac afterload [37]. Subsequent effects on maximum ventricular +dP/dt and peak pressure, increased ventricular end-systolic volume, and decreased ventricular ejection fraction in the developing heart indicate reduced cardiac function associated with slower embryonic growth rate. Interestingly, it is unclear whether the increased peripheral resistance of the CAM associated with hypoxia results from active vasoconstriction, or from increased vascularization. Hypoxia in early development has been demonstrated to increase both expression of HIF1-α and VEGF in the CAM [38], as well as CAM vascularization [39], even as hyperoxia decreases vascular growth [40]. Local regulation of CAM blood flow, presumably by vasomotion, occurs in the CAM at least by Day 15 approximately [41], and is likely to occur much earlier. However, specifically neurally mediated effects on CAM blood flow early in development are unlikely, since cardiac and other reflexes appear in the last few days of development in the avian embryo [42].

Another method that has been used to alter afterload early in development of the avian embryo is experimental banding of the outflow tracts of the embryonic heart. This can be achieved by either applying a clip to the outflow tract, or by placing a ligature around the outflow tract and tightening it to various extents, proportionately increasing resistance to blood flow and increasing afterload. Outflow tract banding and the associated increased afterload alter myocardial architecture and, in turn, cardiac hemodynamics of the early chicken embryo [28,43,44,45,46]. The net effect is an alteration in the gross morphology of the developing heart.

Volume loading has also been used as a mechanism for increasing afterload in early chicken embryos [43,47,48,49,50]. Interestingly, such experiments have revealed adaptation in right and left ventricle passive properties to chronically altered mechanical loading conditions, as well as significant contractile reserve in early cardiac development [47]. Of the various methods that have been used to alter afterload in the chicken embryo, volume loading is potentially the most complex to interpret, because both afterload and preload may be changed, depending upon the compliance of the vascular beds and other factors. Moreover, volume loading will of course dilute the blood, potentially altering blood viscosity blood oxygen transport characteristics as well as preload and afterload. Future studies that compare the effects of banding and volume loading may further validate each methodology.

#### 2.2.3. Shear Stress

Shear stresses produced by alterations in blood flow, especially at points of curvature or bifurcation, can be independent of blood pressure effects per se. Influences of changes in shear stress associated with blood flow per se in the avian embryo influence both cardiac development [34,35,51,52,53,54] as well as maturation of cardiac valves [55] and central vessels, including the aorta [56,57,58,59]. The mechanism likely involves the altered expression of mechanosensitive genes that are involved in cardiovascular development and growth. For example, experimental clipping of the right lateral chorioallantoic vein in Hamburger Hamilton stage 17 embryos decreases cardiac expression of endothelin-1 (ET-1) and upregulates lung Krüppel-like factor 2 (KLF2) and nitric oxide synthase (NOS-3), genes that are sensitive to changes in shear stress [51] and are important for normal development.

Shear stress effects in the CAM itself have also been investigated, where vascular adaptation to shear stress, including the formation of microvascular collaterals following vascular clipping, occurs in Hamburger Hamilton stage 40 chicken embryos [60,61]. Moreover, arterial-venous differentiation and formation of the arterial tree within the CAM are dependent in part upon blood flow and the local shear stresses that derive from this flow [62].

### 2.3. The CAM as Model Vascular Bed for Studying Angiogenesis

The CAM has been a highly useful model for exploring general mechanisms of angiogenesis and arterial tree formation (Figure 1C) [3,38,63,64,65,66,67,68]. An example of a specific use of the CAM to study the effects on vascular bed formation is the application of compounds with angiogenic or antiangiogenic properties; for example, see [4,63,66,69]. These compounds include the angiogenesis-stimulating fibroblast growth factor (FGF), the vascular endothelial growth factor (VEGF), and the angiopoietins, or antiangiogenic factors that typically act by inhibiting angiogenic factors, such as tyrosine kinase inhibitors and VEGF pathway antibodies. Compounds affecting angiogenesis can be applied topically in ovo through a small hole inserted in the eggshell, or topically applied to the CAM in embryos grown ex ovo. Findings from such studies have been highly useful in elucidating process in angiogenesis and pathological conditions that alter it, as elucidated in several excellent reviews [63,66,67,69].

### 2.4. The CAM as an Assay Tool

Aside from the CAM’s own interesting biology and its utility in understanding cardiovascular development, another theme in CAM research is the exploitation of this membrane and its vasculature as an inexpensive, tractable vascular bed for use in a wide variety of assays (Figure 1D) [69]. For example, the CAM assay has been employed in studies assessing drug delivery [70,71,72,73] and toxicological susceptibility [69,74]. The CAM model has also proven effective in assaying nanoparticle characterization associated with drug delivery [75,76,77,78]. Additionally, the CAM has also proven useful in xenobiotic preparations exploring tissue grafts and tumor growth and metastasis, where tumor tissue is grafted into the CAM where the CAM vasculature infiltrates and supports the tumor tissue as it grows; for example, see [65,79,80].

### 2.5. The CAM as an Accessible Vascular Bed

**Figure 3:**
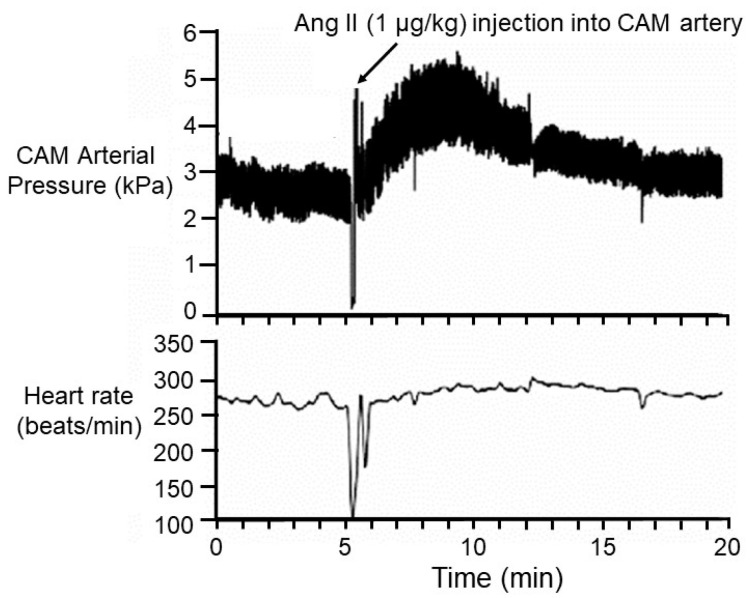
Arterial blood pressure traces and calculated heart rate in a Day 21 chicken embryo before and after injection of the hypertensive agent, angiotensin II. Blood pressure was measured in a chronically indwelling cannula that had been implanted in a CAM artery by keyhole surgery through a small hole in the eggshell. CAM arterial blood pressure, assumed to be a proxy for systemic embryonic pressure, increased dramatically within less than a minute after injection, and stayed elevated for at least 15 min. Heart rate was largely unchanged. These data indicate that receptors for ang II are present at hatching, typically occurring on Day 21 post-fertilization. Modified from [83].

The CAM lies just under the eggshell, allowing its major vessels to be easily accessible through keyhole surgery. As such, the CAM has been the site of choice (sometimes the only practical choice) for accessing the general circulation for blood sampling or drug or dye injections (Figure 1E); for example, see [30,33,34,81,82,83,84,85,86]. For instance, numerous studies have injected cholinergic and adrenergic agonists or antagonists into a chorioallantoic artery or vein at various stages of chicken embryonic development to map the developmental progression of the appearance of receptors, innervation, and secretory organs; for example, see [81,85,86,87,88]. Figure 3 shows a typical protocol, where arterial blood pressure is measured in a CAM artery before and after injection of an agonist. In this example, the ang II heart rate is typically also recorded, and mean values for each parameter are often calculated.

Notable is that the ease of access to discrete arteries or veins is a function of the embryonic stage. In the earliest stages (up to 4 or 5 days post-fertilization), each major artery perfusing a region of the CAM lies very closely beside, above, or below the corresponding vein serving that region of the CAM. Indeed, the two vessels often adhere to each other. Thus, while hints of vessel identity in early embryos can be gleaned by differences in color or, given the right illumination under high power, the direction of blood flow, generally it is not possible to assign identity to specific arteries or veins without vessel dissection in the earliest stages of CAM development. Consequently, in this article, we mostly use the general term “vessels” because, in fact, changes in vascular architecture or distribution are likely to be closely mirrored in arteries and veins because of their strong tendency for adjacent co-location.

### 2.6. Untested Assumptions Regarding the CAM Vasculature

An assumption being made in many studies within the themes of CAM vasculature research that we have outlined above is that there are no special characteristics of the CAM that would introduce complexities in translating these studies into clinical situations, thus allowing the findings on CAM physiology, morphology, and molecular biology to be translatable to mammalian systems. Yet, two key questions have not, to our knowledge, been adequately addressed to unreservedly recommend the CAM as a model vascular bed. The first is the following: “Just how representative is the CAM as a vascular bed?” While the CAM is only analogous to the other gas exchange organs (lungs, gills), we know that often the characteristics, including neural and endocrine regulation, of the gas exchange vascular beds are qualitatively different from systemic vascular beds. Thus, for example, lungs are characterized by cholinergic vasoconstriction, whereas skeletal musculature is typified by cholinergic vasodilation. CAM capillary ultrastructural characteristics—no basement membrane, few organelles, primarily mitochondria and rough endoplasmic reticulum—further support the CAM vasculature as a reasonable model for structurally similar tumor vessels [89]. Notable, however, is that physiological experiments isolating the CAM for the embryonic systemic circulation and perfusing the CAM’s vascular bed with agonists and antagonists, a classic pharmacological approach that has been applied to numerous other vascular beds to characterize specific vasomotor responses and receptor populations, have not been applied to the avian CAM. Notably, however, isolated arterial rings from the CAM were exposed to several vascular agonists and antagonists, revealing many similarities to the fetoplacental arteries of mammals [90], which differ in substantial ways from typical systemic vascular beds. For example, fetoplacental vessels lack autonomic innervation [91], unlike systemic vessels. Additionally, in a comparison of embryonic femoral arteries to CAM arteries, CAM arteries lacked a ß-adrenoceptor-mediated contraction, and hypoxia increased the sensitivity of femoral arteries but not CAM arteries to the ß-adrenoreceptor agonist isoproterenol [84].

The lack of a complete characterization of the CAM’s pharmacology and how it changes during its development and senescence raises a second key question: “What is the relative amount of vascularization (measured, for example, as number or length of vessels or their collective cross-sectional area) of the CAM vs. the embryonic body per se in early development?” As indicated above, the CAM is located both in series and in parallel with the systemic vasculature (Figure 2). Thus, changes in blood pressure in the CAM directly affect the blood pressure in the systemic circulation of the embryo’s body, and vice versa, because of the intimate connection between vascular beds. Typically, cardiovascular reflexes (e.g., baroreflexes) of bird embryos are assessed by cannulating an artery in the CAM [81,85] and measuring heart rate and blood pressure. Consider the hypothetical situation in which the volume and cross-sectional area of the CAM more or less matches that of the embryo’s systemic vasculature (again, this has not to our knowledge been assessed). An experimenter injects systemic vasoconstrictor into a CAM vessel of an avian embryo, waits for it to circulate, and records the finding of no blood pressure change in response to the injection. The experimenter might conclude that, at that particular developmental stage, there are no receptors present to mediate the response. However, in what would be the ultimate cardiovascular developmental irony, the lack of a blood pressure response could also have arisen because the systemic vessels may have vasoconstricted even as the CAM vessels vasodilated, with the hypertension in the systemic circuit cancelling, in the eyes of the observer, the hypotension in the CAM gas exchange circuit! Clearly, if we are to assume the CAM is a representative systemic vascular bed, we need to verify that in future experiments.

## 3. A Case Study in Vasculature Analysis of the Chicken CAM

Having discussed some of the themes in CAM research, we now turn to presenting a case study examining the influences of toxicants on the CAM vasculature, reflecting some of those major themes.

### 3.1. Patterns of Normal Vasculature Growth

**Figure 4:**
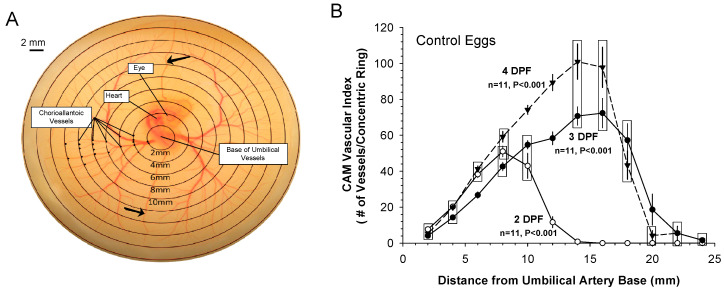
Vascular index of the CAM. (**A**) A commonly employed methodology for determining a vascular index for the chorioallantoic membrane of the chicken embryo grown ex ovo involves digital analysis of images of the living CAM. Variations on this methodology have been long established for both chicken and alligator embryos [40,92,93]. Embryos are first grown ex ovo on yolk in glass dishes, which provides for normal development of the circulation superficially on the yolk [4,94], allowing for easy observation of the vasculature. This photo of the CAM of a 3-day-old embryo was digitally superimposed upon its concentric rings centered on the base of the umbilical artery, and extending out every 2 mm past the edge of the growing CAM. The vascular index is calculated from the number of visible blood vessels (not discriminating between arterial or venous vessels) that intersect any point on each concentric ring. No distinction is made between arteries and veins in this analysis, as the two vessels are usually visually indistinguishable without surgical intervention at this early stage of development. (**B**) Developmental changes in in vivo vascularization in the chicken embryos from Day 2 to Day 4 is revealed by a plot of vascular index as a function of distance from the embryo. Mean values ± SE are shown. *n*= 11 for each point for each day. Arrows indicate superior and inferior vessels subjected to additional quantification in Figure 4B.

Numerous studies have investigated angiogenesis in the chicken embryo’s CAM, using a variety of evaluative instruments and indicators of vascular growth [40,92,93,94] to quantify the vascularity of the CAM through the quantification of a vascular index. Figure 4A shows the methodology used to determine the vascular index, while the normal progression of growth of the major vasculature out from embryo as the CAM forms is illustrated in Figure 4B. The peak vasculature density in Day 2 post-fertilization embryos was at a distance of ~8 mm from the base of the umbilical artery, with this value increasing to ~15 to 17 mm on Days 3 and 4 post-fertilization. The peak vasculature density is characterized by a consistent smaller vessel growth pattern present on the advancing front at the edge of the CAM. The vasculature density rapidly decreases ~2 to 4 mm following the peak as a consistent pattern on all embryos on Days 2–4 post-fertilization. These smaller vessels are the result of the alternated branching patterns (bifurcations and trifurcations, i.e., branching points) of the vitelline vessels [95]. Branching points start ~ to −4 mm from the base of the umbilical artery, increasing in number of points as the CAM grows away from the embryo. The way the growing vessels fill the available space is of particular interest for a better understanding of tissue perfusion.

### 3.2. Vascularity and Cardiac Perfusion

Considerable interest has been directed to the role of the early circulation of the vertebrate embryo [96,97,98,99,100,101]. Early studies in zebrafish larvae suggested that the primary role of the circulation was in stimulating angiogenesis, although more recent studies have challenged this notion, suggesting that, in hypoxia or when active, the circulation is indeed involved in internal convection necessary for gas exchange [98]. In the chicken embryo, ligation of the outflow vessels of the heart in Days 3 and 4 post-fertilization has no significant effect on gas exchange, suggesting that diffusion of respiratory gases suffices in these near microscopic early embryos [97]. These findings in the chicken embryo beg the following question: “What is the role of the early embryonic circulation”? To test the hypothesis that the pulsatile heart beat and associated blood pressure play a role in early angiogenesis, chicken embryos were treated for a 24 h period with a pure bradycardic drug, ZD7288, thus pharmacologically slowing the heart rate (and thus increasing blood pressure and flow pulsatility) [94]. Experimentally induced chronic bradycardia resulted in significantly altered CAM vessel density in the peripheral regions of the CAM compared to controls, suggesting that alterations in heart rate, blood pressure, and blood flow collectively can alter the growth of CAM vasculature, as evident from the vascular index.

### 3.3. Toxicant-Induced Alterations of CAM Vascular Growth

As outlined above, the avian CAM has been used to assess the impact on angiogenesis of a wide variety of toxicants, angiogenic agonists, and antagonists, as well as environmental challenges such as hypoxia. In the last decade, interest has grown in the biological effects of crude oil and its derivatives on developmental processes, likely related to fairly recent major oil spills such as the Deepwater Horizon oil spill in 2010 [102,103]. Studies have been conducted on the general effects of oil on the avian embryo and its circulation; for example, see [104,105,106,107]. Specific to the developing cardiovascular system, crude oil reduced embryonic heart rate and metabolic rate on Day 12 of incubation in the zebra finch (*Taeniopygia guttata*) [108]. However, to our knowledge, no studies have specifically investigated the effects of crude oil on angiogenesis in the avian CAM, which can be exposed to the vapors of oil on the outer surface of the egg shell [104]. Additionally, there is possible contact through capillarity of the oil through the pores of the egg shell due to oil transferred from feathers of brooding birds as well as oiled nesting material. Disruptions to the CAM vasculature could, for example, account in part or in full for the reduction of the metabolic rate observed in the zebra finch [108].

**Figure 5:**
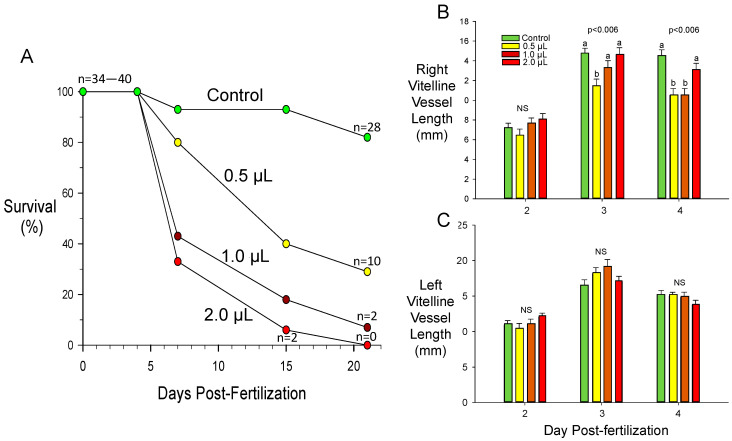
Effects of crude oil on survival and CAM vascular growth in chicken embryos grown in ovo. Doses of crude oil of 0, 0.5, 1, or 2 µL were topically applied to the base of the umbilical artery early in Day 2 post-fertilization. (**A**) Survival curves across all embryonic development. N values of surviving embryos at the start and end of the incubation are indicated (and n at Day 15 for 2.0 µL crude oil exposure). (**B**,**C**) Changes in length of specific posterior and inferior CAM vessels, respectively, as a function of crude oil dose on Days 2, 3, and 4 post-fertilization. Capital letters (**A**,**B**) indicate significant differences between crude oil doses. NS, not significant. Note: Because the arteries and veins of the CAM vasculature typically run very closely together and in parallel to each other, it was not practical to specify a specific type of artery—hence, the general term “vessels” is used. Mean ± 1 SE are presented.

To test the hypothesis that crude oil could affect angiogenesis in the CAM, we pipetted crude oil (0, 0.5, 1, or 2 µL) directly onto the base of the umbilical artery of Day 2 post-fertilization chicken embryos growing ex ovo on yolk in glass dishes [94,109,110,111]. Notably, even this small quantity of crude oil topically applied early on Day 2 resulted in enhanced mortality, on a dose-response basis, across the total incubation period (Figure 5A).

Subsequent to application, we assessed the degree of vascularization later in Day 2, and on Days 3 and 4 using digital imaging and analysis. Using the vasculature index methodology described above, we assessed the effect of oil treatment on the vascular index from the base of the umbilical artery out to 20 mm from the base, in 2 mm increments. Somewhat surprisingly, given the effects of oil in reducing survival, oil treatment at any level had no significant effect upon the vascular index. However, we also measured the lengths of the left and right vitelline vessels from their origin out to the edge of the CAM. Changes in length would be an indicator of tortuosity, the serpentine nature of vessels that is not necessarily indicated by the vascular index. No significant effects (*p* > 0.05) of oil exposure were observed on either right or left vitelline vessel on Day 2 (Figure 5B,C). On Days 3 and 4, however, the overall length of the right vitelline vessels was significantly shortened at intermediate oil doses. However, there was no significant effect (*p* > 0.05) on the left vitelline vessels. These observations indicate regional differences in toxicant sensitivities in vascular beds, even at this early embryonic age. Regional morphological differences associated in the CAM vasculature architecture that are associated with differences in oxygen and water vapor conductance of the overlying eggshell have been identified in the chicken embryo [112]. It would be interesting to investigate whether these morphological differences correlate with different toxicant sensitivities.

### 3.4. Assessing Difference between Vascular Density vs. Vascular Pattern

**Figure 6:**
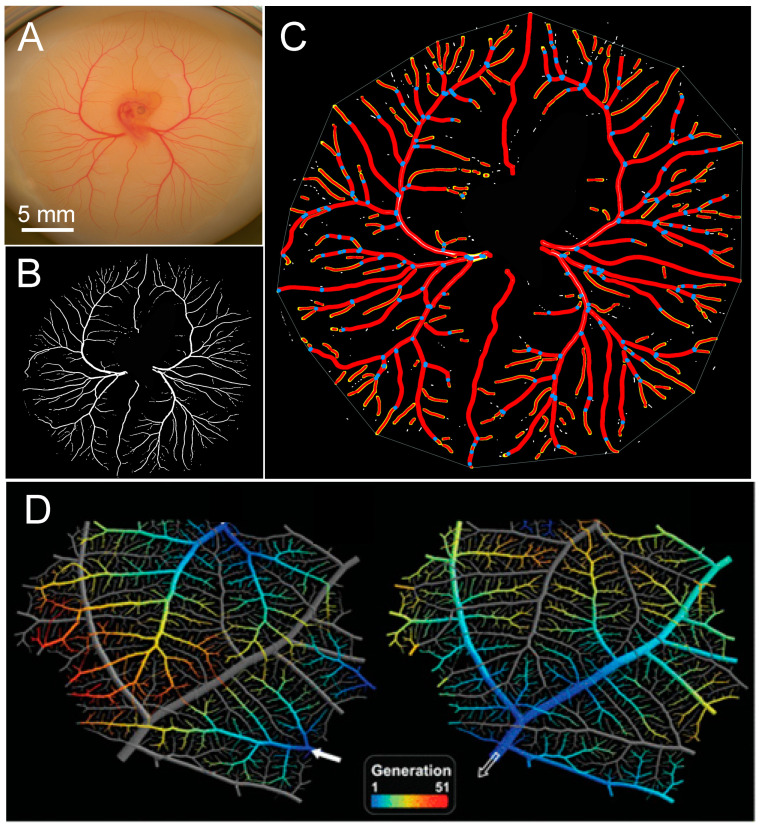
Computer-aided digital analysis of the length and branches of the vessels of the CAM of the 3-day-old chicken embryo. (**A**) A sample image of the CAM of a 3-day-old embryo grown ex ovo. (**B**) CAM vasculature evident in a digitally enhanced image. (**C**) Pathway of the CAM vessels (red lines) and branch points (blue dots). Images in (**B**,**C**) were analyzed using the AngioTool ImageJ plugin. (**D**) Vascular tree topology reconstructed from microscopic images [60]. See text for further details.

**Figure 7:**
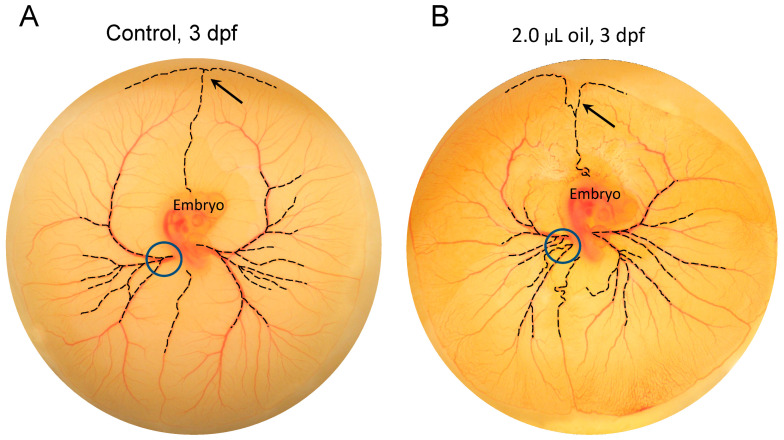
Subtle, reproducible pattern changes in CAM vascular growth induced by low levels of crude oil exposure in the Day 3 post-fertilization embryo. (**A**) In the control embryos, the major vessels at the edge of the CAM branch (enhanced in the image by dashed lines) at nearly right angles (arrow) are shown. In these controls, the major vessels of the CAM emerge from a few vitelline vessels (blue circle). In the sham embryos, in which 2.0 µL of chick Ringer was pipetted onto the embryo’s surface at the base of the umbilical vessels, no change in pattern of CAM vascularization is observed compared to the controls. (**B**) To assess the effect of crude oil exposure on CAM vascularization, embryos received 0.5, 1.0, and 2.0 µL doses of crude oil (Source Oil A from the April 10, 2010 Deepwater Horizon oil spill) topically applied to the base of the umbilical arteries. At any dose of crude oil exposure, the major vessels develop a characteristic deep fork immediately after the feeder vessel has split, further from the CAM edge (black arrow). Additionally, multiple major vessels emerged from the embryo (dashed lines, blue circle). Complicating the analysis of overall CAM vascular density, these additional vessels adjacent to the embryo wall do not continue to branch at the same rate as the controls, and as a consequence the peripherally measured vascular index of the CAM generally is not increased in the oil-exposed embryos, even though the vascular pattern itself changes. dpf, days post-fertilization.

Powerful available software such as AngioTool [113], available as an ImageJ plug-in, and WinCam (Wimasis Image Analysis, Córdoba, Spain) can be used to further quantify key aspects of CAM angiogenesis, as can reconstruction of light and scanning electron microscopic images [60]. Figure 6 shows digitally created images of the CAM of an early chicken embryo, allowing automated calculation of additional variables beyond a vascular index, individual or total vessel length, number of branches, total CAM surface area, and numerous other variables. However, what is more difficult to determine are subtle yet reproducible alterations in the pattern of vessel growth that are not easily revealed by standard metrics like length and branching point density. Even small changes in growth pattern, rather than the overall amount of vasculature per se, could reflect temporal or spatial differences in local growth factors [114,115]. An example of such subtle pattern changes was evident in our experiments with crude oil exposure, where the appearance of the growing leading edge of the CAM membrane was routinely altered in oil-exposed embryos compared to controls (Figure 7), even though the absolute amount of vasculature present appears to not be altered. Quantifying this effect—obvious to the eye but not necessarily to the computer—will be important to discern less obvious but potentially still biologically important effects. Thus, future studies will need to be directed at developing high throughput software tools for the assessment of subtle changes in patterns.

## 4. Unanswered Questions and Future Directions

Several unanswered questions are likely to drive, at least in part, future research in angiogenesis using the avian CAM.

(1) How can we quantify subtle differences in patterns of angiogenesis in the CAM, and do they have any biological significance? Reproducible changes in the pattern of CAM vasculature can signal changes in the local chemical environment regulating the growth and branching of CAM vasculature, and should be investigated in more detail alongside absolute levels of vascularization.
(2) How does the amount of vascularization of the CAM compare to the amount of vasculature in the embryo per se (measured by length, numbers, cross-sectional area, or other metrics) as embryonic development progresses? Knowing this information will help cardiovascular physiologists better interpret changes in blood pressure measured in major CAM arteries, for example. Leading on from this question, we have the following:
(3) Is the CAM a representative vasculature bed of the embryo, overall? The CAM is a venerable model for angiogenesis, but it is also a highly specialized vasculature bed. Experiments that compare the compliance, pharmacology, and other characteristics of the CAM to, for example, a typical systemic vascular bed will be important to verify our findings from the CAM, especially in physiological studies.

Finally, from a biological perspective, we have the following:

(4) Is the chicken embryo’s CAM broadly representative of birds? Certainly, the CAM of ducks [116], geese [117], quail [118], and other birds has been investigated, often from a morphological perspective. However, to our knowledge, a comparative study of the morphology, physiology, and molecular biology of the avian CAM has not been completed.

Answering these and many other questions centered on the avian CAM will help us further understand this fascinating structure, and add additional value to it as a model for angiogenesis in other vascular beds.
